# Associations between Predictors of PTSD and Psychosocial Functioning in Veterans: Results from a Longitudinal Assessment Study

**DOI:** 10.1155/2024/9719635

**Published:** 2024-01-11

**Authors:** R. Pearson, C. Mendoza, J. D. Coppin, S. K. Creech

**Affiliations:** ^1^VHA VISN 17 Center of Excellence for Research on Returning War Veterans, Central Texas Veterans Health Care System, Waco, TX, USA; ^2^Dell Medical School of the University of Texas, Department of Psychiatry and Behavioral Sciences, Austin, TX, USA

## Abstract

Impairments in psychosocial functioning are common in veterans, especially in those with significant mental health symptoms. Although available treatments are aimed at alleviating these symptoms, impairments in psychosocial functioning do not appear to be fully addressed. To achieve rehabilitation and full societal participation, there is a need to identify longitudinal associations of both symptoms and functional outcomes which can be targeted in treatment. United States veterans (*N* = 491) of the Iraq and Afghanistan wars were recruited as part of a longitudinal assessment study which examined predictors of postdeployment adjustment. Veterans were assessed at four timepoints over the course of a two-year period. A Bayesian multivariate multilevel model was used to estimate the association of predictors of PTSD (depression, alcohol use, suicidal ideation, and sleep) on psychosocial functioning as encompassed by quality of life (Quality of Life Scale (QLS)) and disability (World Health Organization Disability Assessment Schedule (WHODAS)) scores over time. As female veterans have unique environmental exposures and functional demands, interactions between predictors and gender were included in all models. There was significant overlap between predictors of PTSD and predictors of disability across domains and quality of life. Depressive symptoms and social support emerged as the strongest predictors of psychosocial functioning. Additionally, suicidality and alcohol use emerged as predictors of quality of life, but not disability. As expected, increases in PTSD symptoms predicted increased disability and decreased quality of life. The effect of depressive symptoms on quality of life was more pronounced for male veterans, and the effect of PTSD and alcohol use on quality of life was more pronounced for female veterans. Findings highlight various treatment targets which have the potential to improve symptoms of PTSD and functional outcomes. Findings highlight an opportunity to leverage intervention and prevention efforts focused on decreasing depression and increasing social support to improve trauma symptoms and maximize rehabilitation and functional recovery in veterans.

## 1. Introduction

There has been a growing interest in describing the trajectory and predictors of functional outcomes in order to better understand and measure recovery. Functional outcomes encompass a range of dimensions including physical well-being, social functioning, ability to complete tasks of daily living, meeting various role expectations, and overall quality of life. The association between psychopathology and functional outcomes is complex. Functional disability can predate the onset of psychological symptoms [1] and persist when symptom remittance has been achieved [2], and psychological symptoms and functional outcomes can move in tandem, exerting a bidirectional influence [3].

Trajectories of functional outcomes have a high degree of variability in the veteran population [4], although a worsening course might be common, especially for female veterans [3]. Further, functional deficits appear to increase the risk for suicide and overall mortality independent of psychological symptoms [5]. Importantly, evidence suggests that psychosocial rehabilitation may be a potential avenue for decreasing suicidal ideation, as employment, meeting basic needs, self-care, living stability, social support, spirituality, resilience, and self-determination are all longitudinally associated with decreased suicidal ideation [6]. Intervention efforts within the Veterans Health Administration (VHA) have traditionally been leveraged at the symptom level, prioritizing evidence-based treatments (EBTs) for diagnosable psychopathology. However, given the ubiquity of functional impairments in veterans and their potential to exacerbate psychological symptoms and increase mortality, there is an untapped potential to intervene at the level of psychosocial functioning to improve treatment outcomes.

To hone targeted intervention, prevention, and outreach efforts at the functional level, predictors of functioning need to be identified. Efforts towards identifying these predictors are underway, with evidence for the influence of mental health symptoms and social factors [7], moral injury [4], and demographic characteristics [8, 9] on functioning. The existing research underscores that contributors to functional outcomes are likely diverse and vary in their degree of malleability. The identification of malleable predictors that can serve as first-line treatment and prevention targets is an important extension of the available research and a logical subsequent course of inquiry.

One approach to identifying these first-line treatment targets is to establish longitudinal predictors that are associated with symptoms *and* functional outcomes. This would allow for the direction of prevention and treatment resources towards those targets which are likely to ameliorate both psychological and functional impairments. Post-traumatic stress disorder (PTSD) should be a priority outcome to examine in conjunction with functional impairment given the high base rate of the disorder in the veteran population and the significant associated emotional distress. Lee et al. [10] recently identified that suicidal ideation, social support, alcohol abuse, and depression were associated with PTSD symptom course over a period of 20 years. There is evidence suggesting that these predictors of PTSD symptom course are also associated with functional outcomes. For example, alcohol use and depression were associated with disability in a sample of veterans with high levels of traumatic brain injury [11]. Further, there appears to be a positive relationship between substance use and functional disability independently of and comorbid with PTSD [12]. Similarly, lack of social support [7, 13] has been associated with dimensions of functional disability in veterans. In addition to the predictors of PTSD course identified by Lee et al. [10], sleep difficulties are another potential driver of both PTSD and psychosocial functioning. Sleep difficulties, including insomnia, sleep apnea, and parasomnias, are very common in veterans [14]. Insomnias and other sleep disorders have been associated with poor functional outcomes in multiple domains, including social functioning, occupational attainment, and quality of life [15, 16]. Sleep difficulties, including disrupted REM sleep and predeployment nightmares, predict the onset of PTSD symptoms over time [17–19]. Further, there is evidence that disrupted sleep (e.g., nightmares) within PTSD is a driver of impairment in psychosocial functioning [20, 21].

Despite these reported associations, there are several limitations to the available evidence. Although the influence of predictors of PTSD course on functional outcomes has been examined in isolation, there is a need to examine these predictors in one model to establish their independent contributions to functional outcomes. Further, much of the available evidence is cross-sectional (e.g., [22]); however, as the association between predictors and functional outcomes is dynamic, their relationship should be examined longitudinally. Additionally, research effort needs to be focused on examining multiple dimensions of functioning, encompassing both degree of disability (e.g., ability to complete tasks of daily living), as well as more subjective experiences of functioning (e.g., quality of life). Lastly, there have been urgent calls within the VA scientific community to report gender-specific results, as female veterans are the fastest growing subgroup of veterans receiving VA care and uniquely at risk for adverse outcomes [23]. Epidemiological studies indicate that prevalence rates of PTSD are higher in women [24]; however, data from the recent U.S. wars in Iraq and Afghanistan has been more mixed with one study suggesting that combat exposure was related to higher rates of depression and PTSD in women compared to men [25] and another finding of no evidence of gender differences [26]. Given the unique characteristics of female veterans in terms of familial, occupational, and social demands, the influence these predictors exert on functional outcomes may also vary by gender. Thus, there is a need to examine which predictors of PTSD course influence functional outcomes and for whom these predictors might be most salient.

To address the limitations of the existing research, this study leverages a longitudinal cohort study of post-9/11 veterans to examine if the determinants of PTSD symptom course also predict quality of life and disability. Suicidal ideation (SI), social support, alcohol abuse, sleep, and depression, along with PTSD, were entered into a Bayesian model to ascertain their influence on functional outcomes. An additional Bayesian model was developed to ascertain if suicidal ideation, social support, alcohol abuse depression, and sleep also predicted PTSD symptom severity in this sample. We specified results by gender, which is a necessary step towards honing and optimizing gender sensitive outreach, prevention, and intervention efforts. It should be noted that the term “predictor” is used throughout in a statistical sense (i.e., to refer to variables on the right-hand side of a regression equation) and does not necessarily imply a causal relationship or that these variables predict the onset of functional impairments. Instead, the overarching goal of this study is to identify potential targets for intervention and prevention, which could ameliorate functional impairment in the course of PTSD.

## 2. Methods

### 2.1. Participants

Participants were 491 post-9/11 veterans who were deployed in Operation Enduring Freedom/Operation Iraqi Freedom and/or Operation New Dawn recruited at the Central Texas Veterans Health Care System (CTVHCS). Participants self-referred through seeing study advertisements (2.8%) were referred from other studies, or participants (2.8%) were recruited through letters sent to those who met initial study eligibility based on medical record data (94.4%). The response rate to recruitment letters was 11.2% (799/7127). Study inclusion criteria were as follows: (1) ability to comprehend and sign the informed consent forms, (2) ability to complete the structured interviews and self-report assessments, (3) willingness to be contacted for follow-up assessments, (4) stability on psychotropic medications (defined as ≥3 months on a selective serotonin reuptake inhibitor or monoamine oxidase inhibitor, >1 month on an anxiolytic or beta-blocker, and >1 month medication discontinuation or “wash out” for all medications) at the time of the baseline assessment, and (5) stability in psychotherapy (≥3-month stabilization for psychotherapy and ≥1-month psychotherapy discontinuation) at the time of the baseline assessment. These latter two criteria were included to ensure that symptoms assessed during the baseline assessment were due to any underlying psychiatric condition and not due to the effects of starting or stopping medications and/or psychotherapy. Changes in treatment were permissible during the current study, as this reflects real-world practice. Exclusion criteria were as follows: (1) planning to relocate out of the CTVHCS system within four months of protocol initiation; (2) meeting criteria for a diagnosis of schizophrenia, schizophreniform disorder, schizoaffective disorder, delusional disorder, or a manic/hypomanic episode; (3) reporting current suicidal or homicidal risk warranting crisis intervention; (4) reporting symptoms consistent with severe traumatic brain injury (TBI) that interfere with their ability to complete the consent process or assessment (i.e., due to ethical concerns about obtaining informed consent and difficulties with completing the structured assessment); or (5) reporting current non-military-related hallucinations or delusions that cause significant distress and/or impairment. See Table 1 for participant characteristics. At timepoints 2, 3, and 4, 87.8%, 86.2%, and 84.9% of participants were retained, respectively. See Table 2 for additional information on missing data.

### 2.2. Study Design and Procedure

This study used data drawn from a longitudinal assessment study consisting of 4 timepoints which were scheduled approximately at 8-month intervals (month 0, month 8, month 16, and month 24). Research technicians established initial eligibility by phone. Participants who met initial eligibility criteria were scheduled for an in-person assessment, where informed consent was obtained, and interview-based and baseline self-report measures were completed. Consenting participants were included in the study and were reassessed using self-report questionnaires administered through mailed packets or a secure survey platform at the month 8 and 16 timepoints. At the month 24, timepoint participants completed another in-person assessment consisting of follow-up interview-based and self-report measures. When needed, participants received referrals for nonstudy treatment resources. Participants were compensated $200 for completing all study appointments. The study took place between 2014 and 2018 and was approved by the CTVHCS institutional review board.

### 2.3. Measures

#### 2.3.1. Predictors

Alcohol Use Disorders Identification Test (AUDIT) [27] is a 10-item measure which assesses alcohol use. The AUDIT is scored on a 5-point Likert scale, ranging from 0 (never) to 4 (daily or almost daily). Total scores range from 0 to 40, with higher scores reflecting more problematic alcohol use. In this sample, the scale had good internal consistency (*α* = 0.88) and test-retest reliability (*r* = 0.86) and strong concurrent validity with other alcohol use screening measures [28].

Columbia Suicide Severity Rating Scale (C-SSRS) [29] intensity of ideation subscale is a 5-item clinician-administered measure designed to systematically assess and track suicidal ideation. The C-SSRS is scored on a 5-point scale, ranging from 1 (least severe) to 5 (most severe). Total scores range from 0 to 25 with higher scores indicating increased intensity of suicidal ideation. In this sample, the scale had good internal consistency (*α* = 0.90), and the scale is well validated in its ability to detect suicidal behavior and risk. The C-SSRS allows for the assessment of lifetime and current suicidal ideation. The C-SSRS items used in the models were those which measure lifetime suicidal ideation prior to study enrollment. These items were measured at baseline and considered time-invariable (i.e., baseline scores were carried forward for the rest of study timepoints). We chose to include history of lifetime C-SSRS items instead of concurrent C-SSRS items as the base rate of suicidal ideation was very low in the current sample.

The Deployment Risk and Resilience Inventory (DRRI) [30] social support scale is a 15-item measure which assesses postdeployment social support across multiple domains including familial, workplace, and community social support. The DRRI is scored on a 5-point Likert scale, ranging from 1 (strongly disagree) to 5 (strongly agree). Total scores range from 15 to 75, with higher scores reflecting increased social support. In this sample, the DRRI social support scale had good internal consistency (*α* = 0.88) and the scale has been validated for use with OIF veterans [31].

Health-Promoting Lifestyle Profile-II (HPLP-II) [32] sleep question (“indicate the frequency with which you get enough sleep”) was used to assess sleep quantity. Sleep was assessed at scores range from 1 (never) to 4 (routinely). There is cumulating evidence suggesting that single-item sleep measures are a valid alternative to lengthier assessments of sleep [33, 34]. Sleep quantity was assessed at baseline and timepoint 4 and imputed at timepoints 2 and 3.

Patient Health Questionnaire-9 (PHQ-9) [35] is a 9-item measure based on DSM-5 diagnostic criteria used for screening, diagnosing, and monitoring depression. It incorporates DSM-5 diagnostic criteria. The PHQ-9 is scored on a 4-point Likert scale, ranging from 0 (not at all) to 3 (nearly every day). Total scores range from 0 to 27, with higher scores reflecting increased depression severity, and clinically meaningful change is a reduction of scores by 50%. In this sample, the scale demonstrated good internal consistency (*α* = 0.91) and there is strong evidence for criterion and construct validity [35].

PTSD Checklist for DSM-5 (PCL-5) [36] is a 20-item measure on a 5-point scale assessing PTSD symptom severity. The PCL-5 is scored on a 5-point Likert scale, ranging from 0 (not at all) to 4 (extremely). Total scores range from 0 to 80 with higher scores reflecting increased endorsement of PTSD symptoms. A score decrease of 18 points constitutes clinically significant change on the PCL-5 [37]. In this sample, the scale had good internal consistency (*α* = 0.97), and there is evidence for good test-retest reliability and convergent and discriminant validity [38].

#### 2.3.2. Outcomes

Quality of Life Scale (QLS) [39] is a 16-item questionnaire which assesses life satisfaction in 16 areas (mate, physical well-being, relationships with others, social, community, civic activities, personal development and fulfillment, recreation, and independence). The QLS is scored on a 7-point anchored scale, ranging from 1 (terrible) to 7 (delighted). Total scores range from 16 to 112, with higher scores reflecting increased satisfaction in life areas. In this sample, the scale had good internal consistency (*α* = 0.93) and there is evidence for high test-retest reliability (*r* = 0.78 to 0.84) and validity [40].

World Health Organization Disability Assessment Schedule-II (WHODAS) [41] is a 36-item questionnaire which assesses functional disability across 7 domains (understanding and communicating, getting around, getting along with people, life activities, work, participation in society, and self-care) as well as a total score. The AUDIT is scored on a 5-point Likert scale, ranging from 1 (none) to 5 (extremely or cannot do). The complex scoring guidelines were used, where total score is calculated in three steps: (1) summing of recoded item scores within each domain, (2) summing of all six domain scores, and (3) converting the summary score into a metric ranging from 0 to 100 (where 0 = no disability and 100 = full disability). In this sample, the WHODAS had good internal consistency (*α* = 0.98) and there is evidence for high test-retest reliability (*r* = 0.98) and concurrent and construct validity [41].

### 2.4. Analyses

#### 2.4.1. Imputation of Missing Data

To preserve the maximum number of cases, missing predictor and outcome data were imputed using a Bayesian multivariate multilevel model. Missing data was coded into the model as a vector of parameters, and missing data was imputed as a probability distributions for each missing data point. This allowed for the quantification of the uncertainty for each specific imputed data point, and it allowed this uncertainty to be propagated through the other parameters in the model. The depression and PTSD imputation models were each fit as multilevel models with varying intercepts for participant. Time, time since deployment, gender, race, ethnicity, sleep, and social support were used as predictors in the depression and PTSD missing data imputation models. All remaining imputation models were also fit as multilevel models, with (alcohol, social support, and sleep) or without (SI and time since deployment) varying intercepts for participant and the following predictors (except when the outcome was time since deployment which included an intercept only): time, time since deployment, gender, race, ethnicity, PTSD, and depression. Varying intercepts among all of the models were modeled as correlated, as correlations between predictors and outcomes are likely for the same individual. The imputation for time since deployment included an intercept only. For all missing data imputation models except for alcohol, truncated Gaussian families were used. Alcohol scores were very skewed, so a truncated lognormal family with identity link was used instead of Gaussian. Normal (0, 1) priors were used for all predictors, since the outcomes consisted of the centered and scaled variables.

#### 2.4.2. PTSD Outcome Analyses

A Bayesian multivariate multilevel model was fit to covariates and predictors of interest for the outcome PTSD (see supplementary materials for model specifications (available here)). A beta family with a logit link function was used to model PTSD, which was scaled to a proportion of the max value of the PTSD response item. Covariates and predictors used for the PTSD model were time, gender, race, ethnicity, time since deployment (covariates), depression, alcohol, SI, social support, and sleep (predictors). Additionally, interactions for gender and time, gender and depression, gender and SI, gender and social support, and gender and alcohol were specified. Model results are reported as means and 95% uncertainty intervals, which are means and 95% quantiles of the posterior distribution for the parameters of interest.

#### 2.4.3. Primary Outcome Analyses

A single Bayesian multivariate multilevel model was fit to covariates and predictors of interest for the primary outcomes of WHODAS and QLS. This model included several likelihood statements (models) both for the main outcomes WHODAS and QLS, as well as for the predictors for missing data imputation purposes (see above for further detail on handling of missing data and supplementary materials for details on model specifications). Covariates and predictors used for the primary outcome analyses were gender, race, ethnicity, time since deployment (covariates), depression, PTSD, alcohol, SI, social support, and sleep (predictors). In both models, interactions for gender and time, gender and depression, gender and PTSD, gender and SI, gender and social support, and gender and alcohol were specified. The variables sleep and SI were 4 and 3 level ordinal categorical variables that were fit as continuous variables. The SI variable was originally a measure on the 0-25 scale, but the distribution of this variable was bimodal and zero-inflated, and thus, it was categorized into a 3-level ordinal variable (scores < 11 = 1, scores > 10 and <21 = 2, and scores > 20 = 3). All other continuous variables were centered and scaled by 1 standard deviation, except for the primary outcome variables QLS and WHODAS, which were left on the original scale. For the primary outcome models, normal (0, 10) priors for all predictors were used. As the trend over time for the QLS was nonlinear, a spline for the trend over time and an interaction for the spline of time with gender were used to model the trend in QLS over time. A penalized thin plate regression spline was used where the amount of wiggliness was controlled by a parameter that is analogous to the standard deviation of varying intercepts or slopes. As this parameter approaches zero, the smooth term approaches a straight line, but as the parameter increases, increasing amounts of wiggliness in the smooth term is allowed, similar to the way in which an increasing standard deviation parameter on a varying intercept allows more heterogeneity. This parameter was estimated to be well above zero (16.03 (4.44–42.60) for women and 17.93 (6.36–45.42) for men) in the case for both smooth terms (men and women), which is evidence that the smooth term provides better fit than a simple linear trend.

In the WHODAS model, time was included as a linear predictor. Both models included varying intercepts for participant. The varying intercepts in the two models were modeled as correlated, as correlations between responses on the QLS and on the WHODAS are likely for the same individual. Model results are reported as means and 95% uncertainty intervals, which are means and 95% quantiles of the posterior distribution for the parameters of interest. Gaussian families were used for the primary outcomes QLS and WHODAS. All models were fit in R version 3.6.3 using the brms package version 2.17.0 which uses Stan on the backend.

All models were fit with 4 chains, 4,000 iterations per chain, of which 1,500 were warm-up samples. Convergence was checked visually by inspecting trace plots and via Rhat (a measure of convergence comparing between and within-chain estimates), and all Rhat was less than 1.05. Model fit was assessed visually via posterior predictive checking, as recommended in Bayesian modeling workflow [42]. Posterior predictive checks involve simulating data from the posterior predictive distribution and comparing this to the actual data. Models that fit well should generate data that are similar (though not exactly the same) to the actual data. Plots of posterior predictive checks are included in the Supplementary Material (see Supplementary Figures 1-3).

## 3. Results

### 3.1. PTSD

Predictors associated with PTSD were social support (-0.27 (-0.37, -0.18)), depression (0.47 (0.35, 0.59)), and sleep (-0.31 (-0.41, -0.21)). There was little discernable effect of alcohol (0.08 (-0.05, 0.20)) and SI (0.07 (-0.14, 0.28)) on PTSD, as the 95% uncertainty intervals broadly covered both sides of zero. There was no discernable difference for men vs. women for any of the examined predictors on PTSD, as the 95% uncertainty intervals for all interactions between predictors and gender broadly covered both sides of zero. See Supplementary Table 1 for correlations and model coefficients and Table 3 for predictor and outcome descriptive statistics over time.

### 3.2. Primary Outcomes

#### 3.2.1. Variability and Correlation

The standard deviations of the varying intercepts in the models are a measure of between-person variability, while the standard deviation of the models is a measure of within-person variability. Both within-person variability and between-person variability were high in all models: the between-person variability for the WHODAS model was 12.01 (10.96, 13.16) and for the QLS model was 8.65 (7.84, 9.51), while the within-person variability was 10.52 (10.12, 10.96) and 9.44 (9.07, 9.83), respectively. The correlation between the varying intercept for the WHODAS model and the varying intercept for the QLS model was -0.52 (-0.62, -0.42), indicating that baseline WHODAS was negatively correlated to baseline QLS in participants. See Supplementary Table 2 for correlations coefficients.

#### 3.2.2. WHODAS

**Figure 1:**
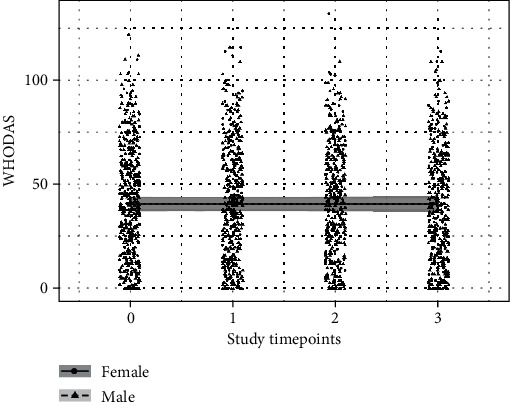
Trajectory of WHODAS scores over time. The figure shows the trend in WHODAS scores over time for men (dashed line) and women (solid line). Lines are mean estimates, and shaded regions are 95% uncertainty intervals.

The baseline (time zero) mean WHODAS score for white, non-Hispanic veterans when depression, social support, time since deployment, and PTSD were at their overall mean value; sleep was at 0 level (poor sleep, the most frequent level); SI was at 0 level (lowest ideation, the most frequent level); and alcohol was at 0 level (least hazardous alcohol consumption, the most frequent level) for women was 40.78 (36.98, 44.61) and for men was 41.03 (38.33, 43.73), which was not discernably different (0.26 (-3.65, 4.15)). There was no discernable trend over time (-0.02 (-0.98, 0.93)) for women and no discernably different trend for men compared to women (-0.06 (-1.09, 0.96)) (see Figure 1). The Bayesian *R*^2^ for the full model was 0.85 (0.84-0.86). There were no discernable differences based on race and ethnicity or time since deployment, as the uncertainty intervals for estimates broadly spanned zero.

**Figure 2:**
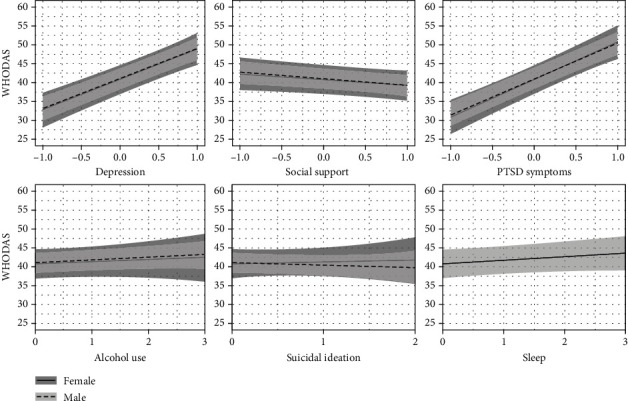
Predictor effects on WHODAS scores. For this figure and Figure 4, depression, social support, and PTSD symptoms are centered and scaled to 1 standard deviation. Alcohol was categorized into a 4-category variable, suicidal ideation a 3-category variable, and sleep a 4-category variable that were fit as continuous variables in the model. The effects on the outcome for each predictor are shown when all other continuous predictors are held at their mean (zero) and alcohol, suicidal ideation, and sleep at their lowest level (zero).

There was little discernable effect of alcohol for women (0.58 (-1.33, 2.48)) or for men (0.74 (-0.42, 1.89)), with no discernable difference in men vs. women, 0.16 (-1.98, 2.24). The same was true for suicidal ideation (0.48 (-2.94, 3.91) for women and -0.59 (-2.95, 1.76) for men, or men vs. women, -1.08 (-5.10, 3.04)) and sleep (0.89 (-0.36, 2.11)) on WHODAS as the 95% uncertainty intervals broadly covered both sides of zero. Depression and PTSD were strong predictors of WHODAS. For every single standard deviation increase in depression, there was a 8.12 (5.81, 10.45) point increase in WHODAS for women and a 7.9 (6.34, 9.5) point increase in WHODAS for men, with no discernable difference for men compared to women, -0.22 (-2.76, 2.32). For each standard deviation increase in PTSD, there was a 9.90 (7.42, 12.42) point increase in WHODAS for women and a 9.43 (7.69, 11.21) point increase in WHODAS for men, with no discernable difference for men compared to women, -0.47 (-3.14, 2.27). Social support was also predictive of WHODAS scores; for each standard deviation increase in social support, there was a 1.53 (0.08, 3.12) point decrease in WHODAS for women and a 1.65 (0.56, 2.76) point decrease in WHODAS for men, with no discernable difference for men compared to women, -0.12 (-1.89, 1.62). Model coefficients are presented in Supplementary Table 2, and the effects of predictors and gender on WHODAS scores are presented in Figure 2.

#### 3.2.3. QLS

**Figure 3:**
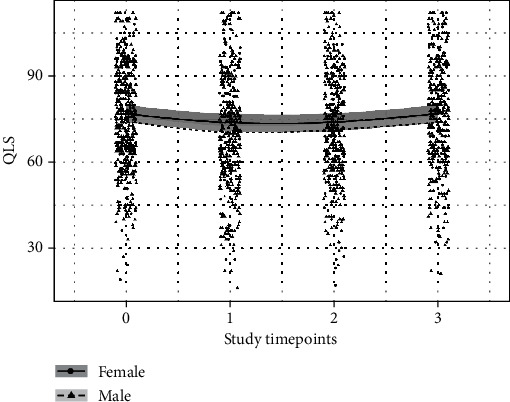
Trajectory of QLS scores over time. The figure shows the trend in QLSS scores over time for men (dashed line) and women (solid line). Lines are mean estimates, and shaded regions are 95% uncertainty intervals.

The baseline (as defined above for WHODAS) mean QLS score was 75.45 (72.59, 78.34) for women and 72.52 (70.5, 74.58) for men; the baseline QLS was slightly less for men compared to women, -2.93 (-5.67, -0.16). The trend over time was nonlinear, as can be seen in Figure 3. The Bayesian *R*^2^ for the full model was 0.74 (0.72-0.76). As with WHODAS, race, ethnicity, and time since deployment had no discernable effect on QLS in this model.

**Figure 4:**
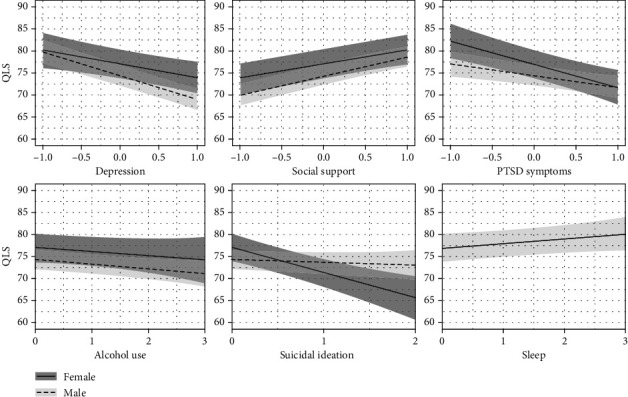
Predictor effects on QLS scores.

Depression, PTSD, social support, and SI were all strongly predictive of QLS. For every single standard deviation increase in depression, there was a 3.04 (0.99, 5.14) point decrease in QLS for women and a 5.35 (3.94, 6.77) point decrease in QLS for men. Men had an additional 2.31 (0.02, 4.58) point decrease beyond that of women. For each SD increase in PTSD, there was a 5.24 (3.05, 7.39) point decrease in QLS for women and a 2.64 (1.07, 4.22) point decrease in QLS for men. The effect of PTSD on QLS was not as great in men, as indicated by the contrast between the slopes of men versus women, 2.60 (0.37, 4.88). For each SD increase in social support, there was a 3.30 (1.87, 4.69) point increase in QLS for women and a 4.27 (3.28, 5.26) point increase in QLS for men. There was an additional 0.97 (-0.58, 2.50) point increase for men vs. women, although this latter estimate is inconclusive as the lower 95% UI covers 0. For each SD increase in SI, there was a 5.58 (2.93, 8.23) point decrease in QLS for women and a 0.61 (1.17, 2.39) point decrease in QLS for men. The contrast between the slopes for men versus women was 4.97 (1.85, 8.07), indicating that there was no appreciable effect of SI on QLS for men. There was some evidence, though not at the 95% level, that increased alcohol predicted decreased QLS in women (-0.91 (-2.49, 0.67)) and men (-1.03 (-2, 0.06)) with little discernable difference for men vs. women (-0.12 (-1.83, 1.60)). Model coefficients are presented in Supplementary Table 2, and the effects of predictors and gender on QLS scores are presented in Figure 4.

## 4. Discussion

This study examined if PTSD and identified predictors of the course of PTSD in veterans, namely, depression, social support, sleep, alcohol use, and SI, also predicted functional outcomes in a longitudinal sample of 491 post-9/11 veterans. As female veterans have unique environmental stressors and functional demands, differential effects of predictors were examined by veteran gender. In this study, the course of disability was relatively stable over time, which aligns with reports of relative invariability in psychosocial functioning, including entrenched functional impairments in large subsets of this population [4]. In contrast, the course of quality of life was more variable, which is expected as these experiential measures of well-being are more likely to be state dependent [43].

Depressive symptoms, social support, and sleep were the strongest predictors of PTSD symptom severity in this sample. Results indicate that previously established predictors of the course of PTSD also predicted disability and quality of life in this sample. Depressive symptoms and social support also emerged as the strongest predictors of functional outcomes in this study, which converges with their predictive power in Lee et al. [10] examination of the course of PTSD. Additionally, SI (in women) and alcohol use emerged as predictors of quality of life, but not disability. Although chronic alcohol abuse is associated with significant functional impairments in all domains [44], alcohol use of this severity level was rare in this sample. Therefore, it is possible that the increases in alcohol use reported here are not severe enough to cause disability but still cause fluctuations in quality of life. As expected, increases in PTSD symptoms predicted increased disability and decreased quality of life. Although the predictive value of trauma symptomatology on disability [45] and quality of life [46] has been widely reported, these findings demonstrate that this association remains significant after controlling for predictors of psychosocial functioning, giving us insights into the unique and potent effect of PTSD on these functional domains.

The interaction between predictors and gender was especially pronounced for quality of life, with the effects of alcohol use on quality of life strongest for female veterans. Research has previously found gender differences in the harms associated with alcohol use: women who use alcohol, even at more moderate levels, are more likely to suffer adverse physical consequences [47], report relationship difficulties in certain contexts [48], and have lower levels of life satisfaction [49]. Additionally, the effect of depressive symptoms on quality of life was more pronounced for male veterans, and the effect of PTSD on quality of life was more pronounced for female veterans. These findings dovetail with research suggesting that depressive symptoms may especially coincide with functional declines in men [50, 51]. There is some evidence that women experience greater functional impairment following trauma exposure [52], although this has not been consistently described [45]. Further research is needed to understand this pattern; however, it could be that the observed gender differences in depression, PTSD, and quality of life may be attributable to societal constructions of gender and gender roles that influence and exacerbate the effects of specific types of symptoms on quality of life [53]. Although stigma can be experienced by anyone with mental health difficulties, it could be that the societal gender roles exacerbate the influence of stigma on depression in men and PTSD in women. This pattern could also be due to the inherent confound between gender with severity and types of traumas that influence depression, PTSD, and quality of life. For example, experiences of sexual trauma are strongly linked to PTSD and highly prevalent among female veterans [54]. We did not examine the type of index trauma or sexual assault exposure; however, it could be that these findings are picking up on a difference in impact on quality of life that is driven by a higher rate of sexual assault exposure among the women in this sample and unmeasured direct and indirect effects of assault on quality of life (Maguen, Cohen, Ren, Bosch, Kimerling, & Seal). Given the pattern of these results, including gender and sexual assault history in future research, examining quality of life in veterans is warranted.

For post-9/11 veterans, the majority of VA mental health resources are directed towards the treatment of PTSD [55]; however, these results suggest that other clinical dimensions significantly impact functional outcomes independent of PTSD. Diversifying treatment targets could increase the potency of treatments offered within the VA. Importantly, these treatment targets are not routinely included in treatments for veterans, even when they are present. For example, severe depressive symptoms in veterans presenting to specialized PTSD treatments within the VA are common [56]; however, EBTs for PTSD do not explicitly address depressive symptoms, and residual depression symptoms are endorsed at high rates after termination of PTSD-focused treatment [57, 58].

Social support is an established risk factor for the emergence and exacerbation of mental health symptoms, and attempts have been made to intervene at the level of social functioning for an array of mental health disorders [59]. Importantly, there is evidence that social support could be an effective addition to the treatment for PTSD [60], and trials including peer support into EBTs for PTSD are underway [61]. Similarly, there have been calls for a greater emphasis on depressive symptoms in the treatment of PTSD, along with a growing recognition that depressive symptoms exist partly independent of trauma symptoms [62, 63]. Available transdiagnostic treatments have grown exponentially over the last decade [64, 65], and dual diagnosis interventions, mostly for comorbid substance use disorders, are gaining ground within VA care [66]. Depressive symptoms appear to be associated with both PTSD and functional impairment within the context of PTSD and independent of PTSD. Given these findings including additive treatments for depressive symptoms in EBTs for PTSD may be merited. For example, behavioral activation (BA), an effective treatment for depression, has been successfully leveraged for the treatment of PTSD [67], with reported downstream effects for comorbid depressive symptoms. Including BA in PTSD treatment protocols may be relatively seamless given the behavioral focus of these protocols and is potentially an untapped means to increase treatment effects and decrease functional disability. Ultimately, adding these treatment components may be most beneficial for those individuals who have severe functional impairment or for those who are at risk for treatment nonresponse. There is a need for further research to evaluate what would be the optimal method and timing for incorporating any additional treatment components into PTSD treatments and who might benefit most from them.

The current study has several strengths. First, as veterans were followed longitudinally, functional outcomes and the effects of predictors were mapped over time. Our sample size was also large, lending confidence to results. In addition, we were able to nuance results by measuring multiple dimensions of psychosocial functioning and modeling effects by gender. Despite suggesting various promising avenues of future research, there are also certain limitations to the current study. The effect of SI and alcohol use on PTSD symptom severity was relatively weak. This is likely partially the result of floor effects in this nonclinical sample. That is, the severity and variability of psychopathology (i.e., PTSD, alcohol use, and SI) were relatively low in this sample, and therefore, the strength of effects may have been constrained. This may have been especially pertinent for SI, and lifetime SI was used for the current study, as current SI was very rare in this sample. Future studies should oversample veterans with diagnosable psychopathology and psychosocial impairment to investigate whether results generalize to these veterans and to further examine the associations between predictors with relatively low base rates in this sample (i.e., current SI) and psychosocial outcomes.

This study only included veterans who are enrolled in VA services, and therefore, results may not generalize to the broader population of veterans. We also relied exclusively on self-report measures of predictors and functional disability, and our methodology would have been strengthened by the inclusion of other measurements, such as ecological momentary assessment, medical record data (i.e., service connection and health status), or corroborating family assessments. This might be especially pertinent for sleep and SI, which were not associated with disability and/or quality of life in this study. This is surprising given the body of research associating sleep and SI with functional impairments [68, 69]. Sleep was only assessed at baseline and timepoint 4. Our models took into account the uncertainty inherent in the imputed data and propagated this uncertainty to the other model parameters; however, future research would be strengthened by the inclusion of additional measurements of these constructs to ascertain whether this lack of association is spurious or true. Additionally, as functional disability was rather invariable in the current study, a longer follow-up period and/or examining functional disability closer to trauma exposure (e.g., by starting assessment in active duty personnel or recently separated veterans) may provide additional insights into which predictors are associated with changes in disability over time. Lastly, future studies might consider exploring how changes between examined predictors and psychosocial outcomes relate to changes in PTSD symptoms. Examining these relationships could elucidate the sequencing of risk (or convergingly resilience) and suggest potential entry points for intervention.

Despite the limitations, this study contributes to the growing literature examining psychosocial outcomes in veterans. To successfully address veterans' unique and comorbid mental health challenges, an arsenal of evidence-based treatment approaches is likely required. Results suggest that depressive symptoms and social support are treatment targets which can potentially be integrated into existing PTSD interventions. Additionally, stand-alone intervention focused on psychosocial outcomes could be offered to veterans as a lower barrier initial treatment option than EBTs for PTSD. There is evidence that these lower threshold interventions may be an eventual gateway to more focused PTSD treatment [70]. This approach is in line with a broader shift within the VA towards recovery and resilience-based alternatives to traditional treatment options. As available treatment options fail to fully address psychosocial functioning deficits in veterans presenting with PTSD, there continues to be a need to focus research efforts on establishing data-driven priority treatment targets. Results outlined in this study can be used to guide these efforts, with the overarching goal of improving symptoms and facilitating veterans' reintegration and rehabilitation.

## Figures and Tables

**Table 1:** Baseline sample characteristics (*N* = 491).

	Female *M* (SD)	Male *M* (SD)
Age	39.6 (8.3)	41.6 (9.1)
Years in active duty military	10.7 (7.4)	11.5 (7.5)
Education	15.1 (1.9)	14.6 (2.3)
Time since deployment (months)	84.9 (36.2)	90.1 (30.5)
Number of OIF/OEF deployments	1.6 (0.9)	2.1 (1.3)

	Female *N* (%)	Male *N* (%)
Female	120 (24.4%)	371 (75.6%)
Latino	25 (20.8%)	73 (19.7%)
Race		
Black	48 (40%)	102 (27.5%)
White	47 (39%)	212 (57.2%)
Other	25 (21%)	57 (15.4%)
Above clinical cut-off		
AUDIT, female (>2); male (>3)	44 (36.7%)	162 (43.7%)
Alcohol use disorder	13 (10.8%)	58 (15.6%)
PCL-5 (>30)	68 (56.6%)	205 (55.3%)
PTSD	51 (42.5%)	145 (39.1%)
PHQ-9 (>9)	72 (60%)	190 (51.2%)
Major depressive disorder	52 (43.3%)	94 (25.3%)
Traumatic brain injury	49 (40.8%)	195 (52.5%)

**Table 2:** Missing data for predictors and outcomes over time.

Time	1	2	3	4
	% (*N*)	% (*N*)	% (*N*)	% (*N*)
QLS (quality of life)	0 (0)	17 (85)	15 (75)	19 (91)
WHODAS (disability)	0 (0)	14 (69)	14 (69)	16 (77)
AUDIT (alcohol use)	0 (0)	13 (62)	15 (74)	15 (76)
PHQ-9 (depression)	<0.1 (0)	14 (67)	14 (70)	15 (75)
PCL-5 (PTSD)	0 (0)	13 (66)	14 (71)	16 (78)
C-SSRS (suicidality)	1 (3)	1 (3)	1 (3)	1 (3)
DRRI (social support)	<0.1 (0)	17 (84)	15 (74)	18 (87)
HPLP-II (sleep)	<0.1 (0)	100 (491)	100 (491)	18 (88)

**(a):** 

Time	1	2	3	4
	*M* (SD)	*M* (SD)	*M* (SD)	*M* (SD)
WHODAS (disability)				
Female	44.0 (25)	47.5(27.7)	45.5 (28.1)	42.4 (26.9)
Male	42.0 (27)	41.9 (27.6)	42.6 (26.2)	39.0 (26.9)
QLS (quality of life)				
Female	74.4 (16.6)	68.8 (20.3)	69.4 (20.3)	75.3 (19.6)
Male	75.8 (18.3)	70.8 (19.5)	71.8 (18.3)	75.0 (18.7)
AUDIT (alcohol use)				
Female	3.4 (5.2)	3.5 (5.2)	3.5 (5.7)	3.2 (5.3)
Male	5.1 (6.4)	3.9 (5.2)	4.7 (6.4)	4.2 (5.4)
DRRI (social support)				
Female	37.9 (8.5)	34.6 (9.5)	34.9 (10)	36.8 (9.9)
Male	39.0 (8.2)	37.0 (8.9)	36.7 (8.8)	37.4 (8.7)
PHQ-9 (depression)				
Female	11.6 (6.2)	12.2 (7)	11.7 (7.2)	10.7 (6.2)
Male	10.2 (6.8)	10.4 (6.8)	10.4 (7)	9.4 (6.9)
PCL-5 (PTSD)				
Female	34.7 (21)	35.9 (22.3)	36.8 (22.6)	32.6 (21.6)
Male	34.2 (21.6)	33.1 (21.4)	33 (21.2)	30.7 (21.3)

**(b):** 

Time	1			
	*N* (%)			
C-SSRS⁣^∗^	
Female	0 (*N* = 62, 52.1%) 1 (*N* = 47, 39.5%) 2 (*N* = 10, 8.4%)			
Male	0 (*N* = 231, 62.4%) 1 (*N* = 124, 33.5%) 2 (*N* = 15, 4.1%)			

⁣^∗^Lifetime suicidal ideation is time-invariable.

## Data Availability

Pending appropriate institutional approvals, summary, and deidentified data supporting these findings will be shared upon request. The reason is that government regulations prohibit open sharing of data from VA patients in this study.

## Supplementary Materials

Supplementary Materials Additional information on model specifications, model coefficients, and correlations is included in the supplementary materials. Additionally, posterior predictive check plots are included in supplementary materials to elucidate model fit.
