# Correction to “Associations between Predictors of PTSD and Psychosocial Functioning in Veterans: Results from a Longitudinal Assessment Study”

**DOI:** 10.1155/da/9846714

**Published:** 2025-11-15

R. Pearson, C. Mendoza, J. D. Coppin, and S. K. Creech, “Associations between Predictors of PTSD and Psychosocial Functioning in Veterans: Results from a Longitudinal Assessment Study,” *Depression and Anxiety* 2024 (2024): 9719635, https://doi.org/10.1155/2024/9719635.

In the article titled “Associations between Predictors of PTSD and Psychosocial Functioning in Veterans: Results from a Longitudinal Assessment Study,” information was omitted in the Acknowledgments section, and a reference was omitted in error. The corrected Acknowledgments section appears below:

Acknowledgments

Financial support for this study was provided by VA Office of Rehabilitation Research and Development (RR&D) Merit Grant I01RX000304 to Dr. Creech. All of the authors of the manuscript are also supported by the Department of Veterans Affairs VISN 17 Center of Excellence for Research on Returning War Veterans and the Central Texas Veterans Affairs Healthcare System. The authors of this manuscript also acknowledge the contributions of the prior teams of investigators and research assistants who were involved with any phase of VA RR&D Merit Review Award # I01RX000304 (to Meyer, Morissette) and later (to Creech).

The reference is shown below:

Frankfurt, S. B., DeBeer, B. B., Morissette, S. B., Kimbrel, N. A., La Bash, H., & Meyer, E. C. (2018). Mechanisms of moral injury following military sexual trauma and combat in post-9/11 US war veterans. *Frontiers in Psychiatry*, *9*, 520. doi: 10.3389/fpsyt.2018.00520

This should supplement the following sentences in text:

This study used data drawn from a longitudinal assessment study consisting of 4 timepoints which were scheduled approximately at 8-month intervals (month 0, month 8, month 16, and month 24) [1].

We apologize for these errors.

