# Supplementary material for: Associations between Predictors of PTSD and Psychosocial Functioning in Veterans: Results from a Longitudinal Assessment Study
Source: Depress Anxiety. 2024 Jan 11;2024:9719635. doi: 10.1155/2024/9719635 (PMC11918957; doi:10.1155/2024/9719635)
Supplement: Supplementary Materials — Additional information on model specifications, model coefficients, and correlations is included in the supplementary materials. Additionally, posterior predictive check plots are included in supplementary materials to elucidate model fit. [file 9719635.f1.docx]

**SUPPLEMENTARY MATERIAL**

**MODEL RESULTS**

**Supplementary Table 1:** **Correlations and Model Coefficients for PTSD (PCL-5)**

| **Correlation among varying intercepts** | | | | | | |
| --- | --- | --- | --- | --- | --- | --- |
|  | Estimate | Est.Error | l-95% CI | U-95% CI | Bulk ESS | Tail ESS |
| ptsd,alcohol | -0.11 | 0.06 | -0.24 | 0.02 | 1053 | 2221 |
| ptsd,depression | 0.57 | 0.05 | 0.46 | 0.66 | 1210 | 2521 |
| alcohol,depression | 0.06 | 0.06 | -0.05 | 0.16 | 2445 | 4883 |
| ptsd,social support | 0.47 | 0.08 | 0.31 | 0.61 | 1218 | 2445 |
| alcohol,social support | 0 | 0.05 | -0.1 | 0.1 | 3599 | 6225 |
| depression,social support | 0.46 | 0.07 | 0.32 | 0.59 | 1582 | 3473 |
| ptsd,sleep | 0.52 | 0.08 | 0.36 | 0.66 | 889 | 1603 |
| alcohol,sleep | 0.07 | 0.06 | -0.04 | 0.18 | 2466 | 5310 |
| depression,sleep | 0.94 | 0.02 | 0.91 | 0.97 | 1811 | 3844 |
| social support,sleep | 0.16 | 0.06 | 0.04 | 0.29 | 2952 | 5812 |
|  | | | | | | |
| **Fixed effects** | | | | | | |
|  | Estimate | Est.Error | l-95% CI | U-95% CI | Bulk ESS | Tail ESS |
| Intercept | -0.39 | 0.13 | -0.65 | -0.13 | 1776 | 3869 |
| ethnicity_hispanic | 0.12 | 0.11 | -0.09 | 0.33 | 2342 | 4501 |
| gender_male | 0.11 | 0.13 | -0.15 | 0.36 | 2741 | 4661 |
| alcohol | 0.08 | 0.06 | -0.05 | 0.2 | 3462 | 5950 |
| alcohol:gender_male | 0.01 | 0.07 | -0.12 | 0.14 | 4813 | 6970 |
| depression | 0.47 | 0.06 | 0.35 | 0.59 | 3084 | 5750 |
| depression:gender_male | 0.02 | 0.06 | -0.1 | 0.15 | 6675 | 7592 |
| si | 0.07 | 0.11 | -0.14 | 0.28 | 2905 | 5144 |
| si:gender_male | -0.04 | 0.13 | -0.29 | 0.21 | 2821 | 5352 |
| sleep | -0.31 | 0.05 | -0.41 | -0.21 | 1030 | 1990 |
| social support | -0.27 | 0.05 | -0.37 | -0.18 | 2828 | 4979 |
| social support:gender_male | -0.03 | 0.05 | -0.14 | 0.07 | 5937 | 7279 |
| time_deployment | 0 | 0.03 | -0.06 | 0.07 | 3156 | 5712 |
| race_black | 0.15 | 0.09 | -0.04 | 0.33 | 2477 | 4385 |
| race_other | -0.03 | 0.12 | -0.26 | 0.19 | 2579 | 4721 |
| time | -0.01 | 0.03 | -0.06 | 0.05 | 9933 | 7734 |
| time:gender_male | -0.05 | 0.03 | -0.11 | 0.01 | 12412 | 8291 |

**Supplementary Table 2: Correlations and Model Coefficients for WHODAS and QLS**

| **Correlations among varying intercepts** | | | | | | | | |  | |  |
| --- | --- | --- | --- | --- | --- | --- | --- | --- | --- | --- | --- |
|  | Estimate | | Est.Error | | l-95% CI | | U-95% CI | | Bulk ESS | | Tail ESS |
| qls,whodas | -0.52 | 0.05 | | -0.62 | | -0.42 | | 1974 | | 3684 | |
| qls,ptsd | -0.04 | 0.09 | | -0.21 | | 0.13 | | 567 | | 1252 | |
| whodas,ptsd | 0.31 | 0.07 | | 0.18 | | 0.44 | | 755 | | 1998 | |
| qls,alcohol | 0.09 | 0.07 | | -0.04 | | 0.22 | | 780 | | 1774 | |
| whodas,alcohol | -0.12 | 0.06 | | -0.24 | | 0 | | 1059 | | 2613 | |
| ptsd,alcohol | 0.02 | 0.06 | | -0.09 | | 0.13 | | 2375 | | 4742 | |
| qls,depression | -0.17 | 0.08 | | -0.33 | | -0.01 | | 618 | | 1406 | |
| whodas,depression | 0.4 | 0.06 | | 0.28 | | 0.52 | | 855 | | 2442 | |
| ptsd,depression | 0.85 | 0.02 | | 0.81 | | 0.88 | | 4754 | | 7165 | |
| alcohol,depression | 0.06 | 0.06 | | -0.05 | | 0.17 | | 3275 | | 6402 | |
| qls,social support | 0.29 | 0.07 | | 0.15 | | 0.42 | | 1193 | | 2455 | |
| whodas,social support | 0.03 | 0.07 | | -0.11 | | 0.16 | | 1668 | | 4284 | |
| ptsd,social support | 0.51 | 0.07 | | 0.36 | | 0.64 | | 1568 | | 3884 | |
| alcohol,social support | 0 | 0.05 | | -0.1 | | 0.1 | | 3913 | | 6490 | |
| depression,social support | 0.39 | 0.07 | | 0.24 | | 0.52 | | 2085 | | 3922 | |
| qls,sleep | -0.1 | 0.09 | | -0.26 | | 0.07 | | 570 | | 1408 | |
| whodas,sleep | 0.3 | 0.07 | | 0.16 | | 0.42 | | 814 | | 2157 | |
| ptsd,sleep | 0.9 | 0.02 | | 0.85 | | 0.94 | | 1120 | | 2467 | |
| alcohol,sleep | 0.06 | 0.06 | | -0.05 | | 0.17 | | 2942 | | 5468 | |
| depression,sleep | 0.92 | 0.02 | | 0.87 | | 0.95 | | 1032 | | 2136 | |
| social support,sleep | 0.2 | 0.07 | | 0.07 | | 0.33 | | 4075 | | 6707 | |
|  |  | |  | |  | |  | |  | |  |
| **Fixed effects for QLS** | | | | | | | | | | | |
|  | Estimate | | Est.Error | | l-95% CI | | U-95% CI | | Bulk ESS | | Tail ESS |
| alcohol | -0.91 | 0.81 | | -2.49 | | 0.67 | | 4349 | | 7057 | |
| alcohol:gender | -0.12 | 0.87 | | -1.83 | | 1.6 | | 7165 | | 7630 | |
| depression | -3.04 | 1.06 | | -5.14 | | -0.99 | | 4638 | | 5798 | |
| depression:gender | -2.31 | 1.16 | | -4.58 | | -0.02 | | 7007 | | 7785 | |
| ethnicity_hispanic | 0.19 | 1.21 | | -2.2 | | 2.56 | | 5271 | | 6428 | |
| gender | -2.93 | 1.4 | | -5.67 | | -0.16 | | 4854 | | 5972 | |
| Intercept | 75.45 | 1.47 | | 72.59 | | 78.34 | | 3354 | | 5537 | |
| ptsd | -5.24 | 1.12 | | -7.39 | | -3.05 | | 2148 | | 5265 | |
| ptsd:gender | 2.6 | 1.16 | | 0.37 | | 4.88 | | 7064 | | 8045 | |
| race_black | 1.51 | 1.08 | | -0.62 | | 3.62 | | 4859 | | 6842 | |
| race_other | -1.04 | 1.33 | | -3.65 | | 1.5 | | 4872 | | 6311 | |
| si | -5.58 | 1.36 | | -8.23 | | -2.93 | | 4260 | | 6671 | |
| si:gender | 4.97 | 1.6 | | 1.85 | | 8.07 | | 4510 | | 6318 | |
| sleep | 1.08 | 0.56 | | -0.03 | | 2.16 | | 1228 | | 3625 | |
| social support | 3.3 | 0.73 | | 1.87 | | 4.69 | | 4093 | | 6540 | |
| social support:gender | 0.97 | 0.79 | | -0.58 | | 2.5 | | 8998 | | 8062 | |
| time:gender | -1.66 | 7.1 | | -15.59 | | 12.26 | | 13045 | | 8045 | |
| time:gender | -10.11 | 5.98 | | -22.11 | | 1.45 | | 12938 | | 7951 | |
| time_deployment | -0.69 | 0.48 | | -1.64 | | 0.25 | | 4539 | | 6227 | |
| **Fixed effects for WHODAS** |  |  | |  | |  | |  | |  | |
|  | Estimate | Est.Error | | l-95% CI | | U-95% CI | | Bulk ESS | | Tail ESS | |
| alcohol | 0.58 | 0.97 | | -1.33 | | 2.48 | | 5942 | | 7551 | |
| alcohol:gender | 0.16 | 1.06 | | -1.98 | | 2.24 | | 7657 | | 7797 | |
| depression | 8.12 | 1.18 | | 5.81 | | 10.45 | | 5273 | | 6574 | |
| depression:gender | -0.22 | 1.3 | | -2.76 | | 2.32 | | 7642 | | 7789 | |
| ethnicity_hispanic | 0.67 | 1.57 | | -2.4 | | 3.74 | | 4488 | | 6478 | |
| gender | 0.26 | 1.96 | | -3.65 | | 4.15 | | 5498 | | 7329 | |
| Intercept | 40.78 | 1.96 | | 36.98 | | 44.61 | | 4633 | | 6870 | |
| ptsd | 9.9 | 1.27 | | 7.42 | | 12.42 | | 3022 | | 5769 | |
| ptsd:gender | -0.47 | 1.36 | | -3.14 | | 2.27 | | 6648 | | 6965 | |
| race_black | -0.2 | 1.4 | | -2.93 | | 2.53 | | 4740 | | 6376 | |
| race_other | 2.3 | 1.7 | | -1.03 | | 5.65 | | 4718 | | 6525 | |
| si | 0.48 | 1.75 | | -2.94 | | 3.91 | | 3866 | | 6018 | |
| si:gender | -1.08 | 2.07 | | -5.1 | | 3.04 | | 4462 | | 5806 | |
| sleep | 0.89 | 0.63 | | -0.36 | | 2.11 | | 1923 | | 4478 | |
| social support | -1.53 | 0.81 | | -3.12 | | 0.08 | | 4994 | | 7112 | |
| social support:gender | -0.12 | 0.9 | | -1.89 | | 1.62 | | 8483 | | 8262 | |
| time | -0.02 | 0.48 | | -0.98 | | 0.93 | | 10316 | | 8122 | |
| time:gender | -0.06 | 0.52 | | -1.09 | | 0.96 | | 14157 | | 7940 | |
| time_deployment | 0.21 | 0.64 | | -1.05 | | 1.47 | | 4381 | | 6397 | |

**Supplementary Figure 1: Posterior Predictive Checks for QLS***


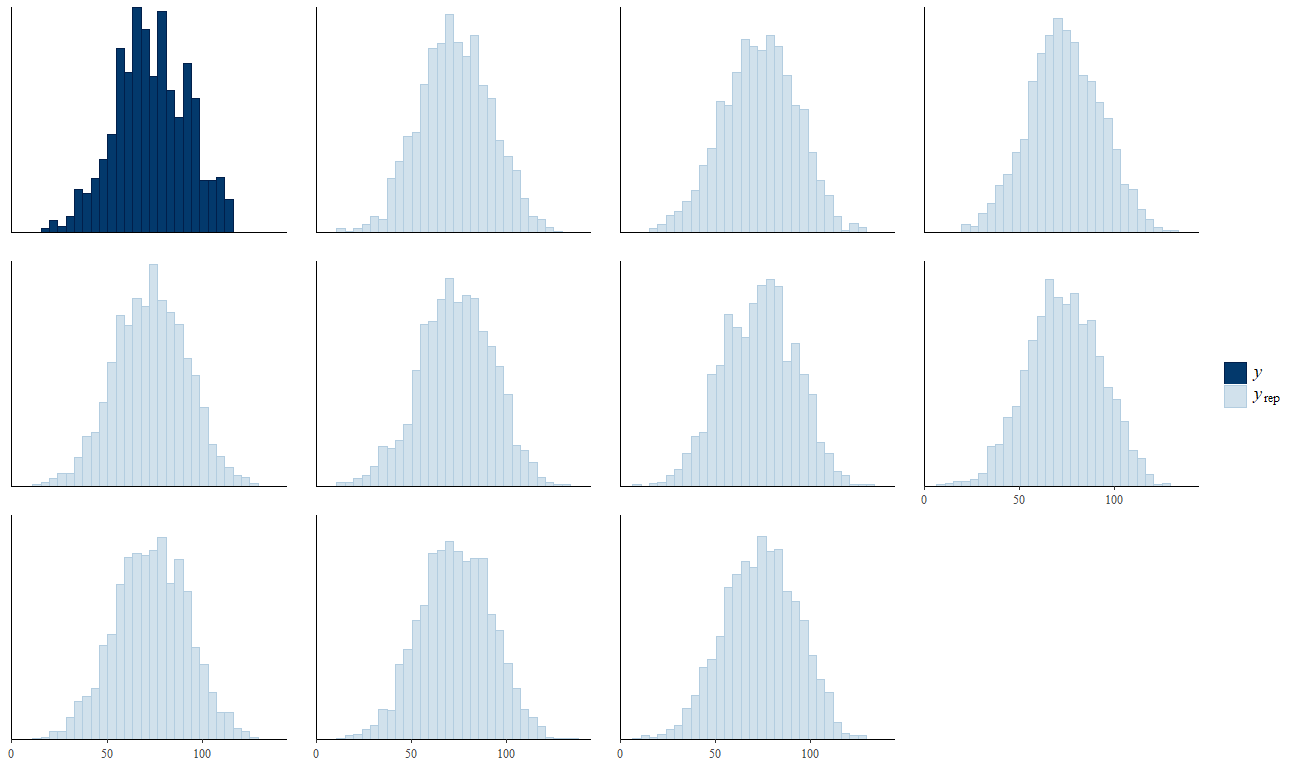


***For supplementary Figures 1-3:** Posterior predictive checks compare simulations of data from the posterior predictive distributions to the actual data. A good generative model should generate data that is similar (but not identical to the actual data. In these plots, the dark colors show the actual data, and light colors the simulations from the model’s posterior predictive distribution. For more information see: Gabry J, Simpson D, Vehtari A, Betancourt M, Gelman A. Visualization in Bayesian workflow. Journal of the Royal Statistical Society Series A: Statistics in Society. 2019 Feb;182(2:389-402.

**Supplementary Figure 2: Posterior Predictive Checks for WHODAS**


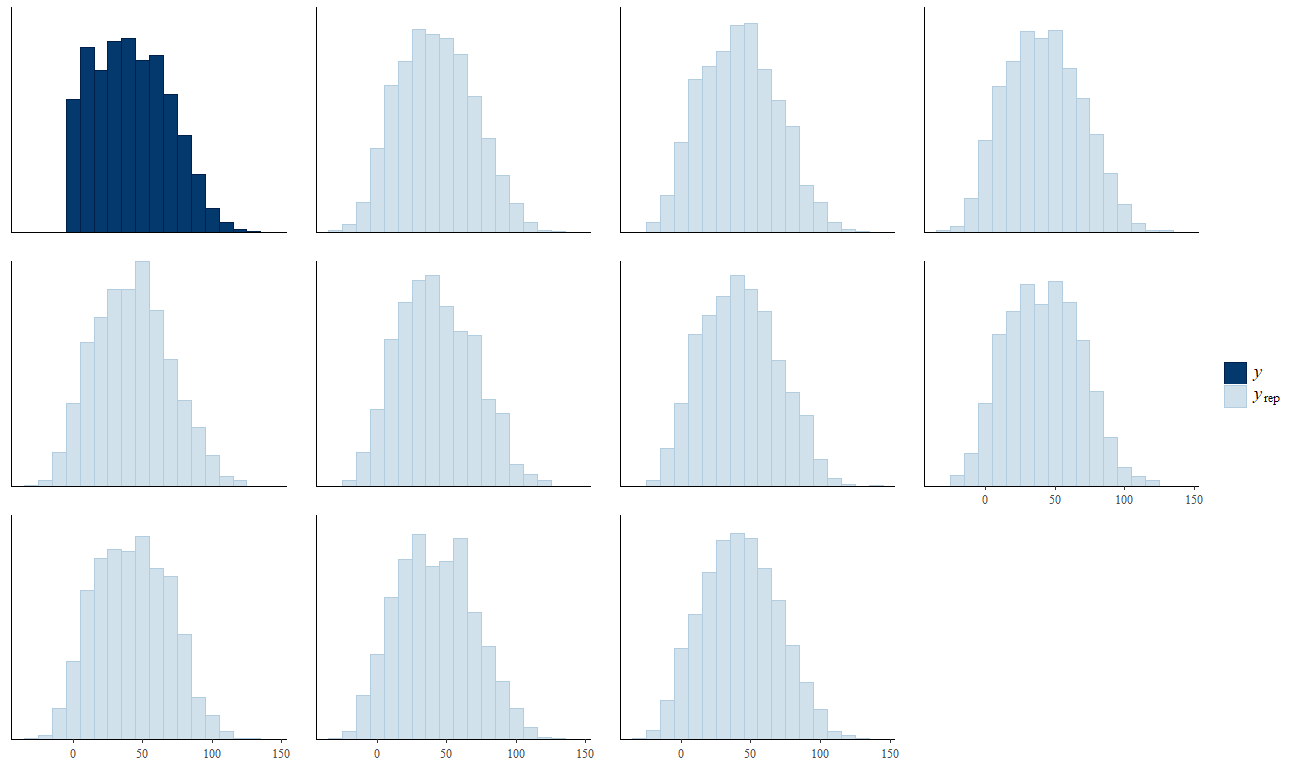


**Supplementary Figure 3: Posterior Predictive Checks for PTSD (PCL-5)**


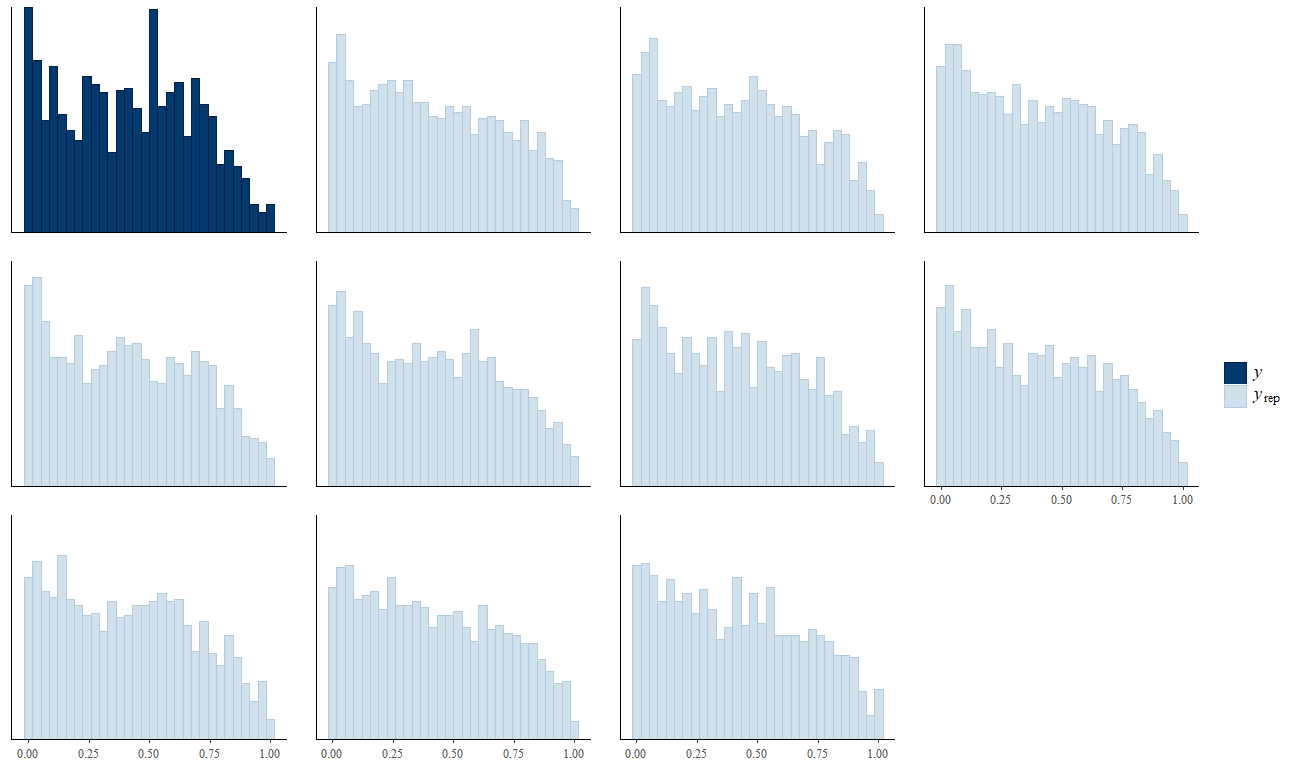


**MODEL SPECIFICATION CODE**

**Model code for WHODAS and QLS.**

Note that results are taken from the “bf_whodas” and “bf_qls” models within the multivariate (i.e. multiple outcome variables model. The other models are for missing data imputation purposes. Some of the models for missing data imputation are truncated models, with truncation points near the upper and lower bounds of the data. This ensures that imputations lie within the range of the actual data.

priors <- c(prior(lkj(2), class=cor),

prior(normal(0,10), class=b, resp="whodas"),

prior(normal(0,10), class=b, resp="qls"),

prior(normal(0,1), class=b, resp="ptsdsymptomsc"),

prior(normal(0,1), class=b, resp="alcohol2s"),

prior(normal(0,1), class=b, resp="silifeca2"),

prior(normal(0,1), class=b, resp="social supportc"),

prior(normal(0,1), class=b, resp="depressionc"),

prior(normal(0,0.5), class=sd, coef="Intercept", resp="ptsdsymptomsc", group='id'),

prior(normal(0,0.5), class=sd, coef="Intercept", resp="alcohol2s", group='id'),

prior(normal(0,0.5), class=sd, coef="Intercept", resp="social supportc", group='id'),

prior(normal(0,0.5), class=sd, coef="Intercept", resp="depressionc", group='id'),

prior(normal(0,0.5), class=sd, coef="Intercept", resp="sleepz", group='id'))

bf_qls <- bf(qls | mi() ~ 1 + gender.f + s(timeM, by=gender.f, k=4) +

mi(depression.c)*gender.f + mi(ptsdsymptoms.c)*gender.f + mi(alcohol)*gender.f + mi(si)*gender.f +

mi(social support.c)*gender.f + mi(sleep) + race.n + ethnicity.n + mi(timedep.c) + (1|p|id)) + gaussian()

bf_whodas <- bf(whodas | mi() ~ 1 + timeM + gender.f + timeM:gender.f +

mi(depression.c)*gender.f + mi(ptsdsymptoms.c)*gender.f + mi(alcohol)*gender.f + mi(si)*gender.f +

mi(social support.c)*gender.f + mi(sleep) + race.n + ethnicity.n + mi(timedep.c) + (1|p|id)) + gaussian()

bf_ptsd <- bf(ptsdsymptoms.c | mi() + trunc(lb=-1.65, ub=2.27) ~ 1 + timeM + gender.f + mi(social support.c) + mi(sleep) + race.n + ethnicity.n + mi(timedep.c) + (1|p|id)) + gaussian()

bf_dep <- bf(depression.c | mi() + trunc(lb=-1.63, ub=2.52) ~ 1 + timeM + gender.f + mi(social support.c) + mi(sleep) + race.n + ethnicity.n + mi(timedep.c) + (1|p|id)) + gaussian()

bf_alc <- bf(alcohol | mi() + trunc(ub=7) ~ 1 + timeM + gender.f + mi(ptsdsymptoms.c) + mi(depression.c) + race.n + ethnicity.n + mi(timedep.c) + (1|p|id)) + lognormal(link="identity")

bf_si <- bf(si | mi() + trunc(lb=-0.5, ub=2.5) ~ 1 + gender.f + mi(ptsdsymptoms.c) + mi(depression.c) + race.n + ethnicity.n) + gaussian()

bf_ss <- bf(social support.c | mi() + trunc(lb=-3.15, ub=1.53) ~ 1 + timeM + gender.f + mi(ptsdsymptoms.c) + mi(depression.c) + race.n + ethnicity.n + mi(timedep.c) + (1|p|id)) + gaussian()

bf_sl <- bf(sleep | mi() + trunc(lb=-0.5, ub=3.5) ~ 1 + timeM + gender.f + mi(ptsdsymptoms.c) + mi(depression.c) + race.n + ethnicity.n + mi(timedep.c) + (1|p|id)) + gaussian()

bf_td <- bf(timedep.c | mi() ~ 1) + gaussian()

m.ptsd.whodas <- brm(bf_qls + bf_whodas + bf_ptsd + bf_alc + bf_si + bf_dep + bf_ss + bf_sl + bf_td +

set_rescor(FALSE),

data = q1b, prior=priors,

control = list(adapt_delta = 0.9999, max_treedepth=13), iter=4000, warmup=1500, init="0", cores=4)

**Model code for PTSD**

Note that results are taken from the “bf_ptsd” model within the multivariate (i.e. multiple outcome variables) model. The other models are for missing data imputation purposes. Some of the models for missing data imputation are truncated models, with truncation points near the upper and lower bounds of the data. This ensures that imputations lie within the range of the actual data.

priors <- c(prior(lkj(2), class=cor),

prior(normal(0,1), class=b, resp="ptsd"),

prior(normal(0,1), class=b, resp="alcohol2s"),

prior(normal(0,1), class=b, resp="silifeca2"),

prior(normal(0,1), class=b, resp="social supportc"),

prior(normal(0,1), class=b, resp="depressionc"),

prior(normal(0,0.5), class=sd, coef="Intercept", resp="ptsd", group='id'),

prior(normal(0,0.5), class=sd, coef="Intercept", resp="alcohol2s", group='id'),

prior(normal(0,0.5), class=sd, coef="Intercept", resp="social supportc", group='id'),

prior(normal(0,0.5), class=sd, coef="Intercept", resp="depressionc", group='id'),

prior(normal(0,0.5), class=sd, coef="Intercept", resp="sleepz", group='id')

)

bf_ptsd <- bf(ptsd | mi() ~ 1 + timeM + gender.f + timeM:gender.f +

mi(depression.c)*gender.f + mi(alcohol)*gender.f + mi(si)*gender.f +

mi(social support.c)*gender.f + mi(sleep) + mi(timedep.c) + race.n + ethnicity.n + (1|p|id)) + Beta()

bf_dep <- bf(depression.c | mi() + trunc(lb=-1.63, ub=2.52) ~ 1 + timeM + gender.f + mi(social support.c) + mi(sleep) + race.n + ethnicity.n + (1|p|id)) + gaussian()

bf_alc <- bf(alcohol | mi() + trunc(ub=7) ~ 1 + timeM + gender.f + mi(ptsd) + mi(depression.c) + race.n + ethnicity.n + (1|p|id)) + lognormal(link="identity")

bf_si <- bf(si | mi() + trunc(lb=-0.5, ub=2.5) ~ 1 + gender.f + mi(ptsd) + mi(depression.c) + race.n + ethnicity.n) + gaussian()

bf_ss <- bf(social support.c | mi() + trunc(lb=-3.15, ub=1.53) ~ 1 + timeM + gender.f + mi(ptsd) + mi(depression.c) + race.n + ethnicity.n + (1|p|id)) + gaussian()

bf_sl <- bf(sleep | mi() + trunc(lb=-0.5, ub=3.5) ~ 1 + timeM + gender.f + mi(ptsd) + mi(depression.c) + race.n + ethnicity.n + (1|p|id)) + gaussian()

bf_td <- bf(timedep.c | mi() ~ 1) + gaussian()

m.ptsd <- brm(bf_ptsd + bf_alc + bf_si + bf_dep + bf_ss + bf_sl + bf_td +

set_rescor(FALSE),

data = q1b, prior=priors,

control = list(adapt_delta = 0.9999, max_treedepth=13), iter=4000, warmup=1500, init="0", cores=4
